# The Optimization of Radiation Synthesis Modes for YAG:Ce Ceramics

**DOI:** 10.3390/ma16083158

**Published:** 2023-04-17

**Authors:** Victor Lisitsyn, Dossymkhan Mussakhanov, Aida Tulegenova, Ekaterina Kaneva, Liudmila Lisitsyna, Mikhail Golkovski, Amangeldy Zhunusbekov

**Affiliations:** 1 Department of Materials Science, Engineering School, National Research Tomsk Polytechnic University, 30, Lenin Avenue, Tomsk 634050, Russia; 2 Department of Radio Engineering, Electronics and Telecommunications, Eurasian National University L.N. Gumilyov, 2, Satpaev Str., Astana 010008, Kazakhstan; dos_f@mail.ru; 3 Department of Solid State and Nonlinear Physics, Al-Farabi Kazakh National University, 71, Al-Farabi Ave., Almaty 050040, Kazakhstan; tulegenova.aida@gmail.com; 4 X-ray Analysis Laboratory, Vinogradov Institute of Geochemistry SB RAS, 1A, Favorsky Str., Irkutsk 664033, Russia; kev604@mail.ru; 5 Department of Physics, Chemistry and Theoretical Mechanics, Tomsk State University of Architecture and Building, 2, Solyanaya Sq., Tomsk 634003, Russia; lisitsyna@mail.ru; 6 Budker Institute of Nuclear Physics, SB RAS, 11, Lavrentiev Ave., Novosibirsk 630090, Russia; golkovski@mail.ru; 7 Department of Technical Physics, Eurasian National University L.N. Gumilyov, 2, Satpaev Str., Astana 010008, Kazakhstan; zhunusbekov_am@enu.kz

**Keywords:** synthesis, YAG:Ce ceramics, structure, high-power electron flux, energy loss, luminescence

## Abstract

Synthesis in the radiation field is a promising direction for the development of materials transformation processes, especially those differing in melting temperature. It has been established that the synthesis of yttrium–aluminum ceramics from yttrium oxides and aluminum metals in the region of a powerful high-energy electron flux is realized in 1 s, without any manifestations that facilitate synthesis, with high productivity. It is assumed that the high rate and efficiency of synthesis are due to processes that are realized with the formation of radicals, short-lived defects formed during the decay of electronic excitations. This article presents descriptions of the energy-transferring processes of an electron stream with energies of 1.4, 2.0, and 2.5 MeV to the initial radiation (mixture) for the production of YAG:Ce ceramics. YAG:Ce (Y_3_Al_5_O_12_:Ce) ceramics samples in the field of electron flux of different energies and power densities were synthesized. The results of a study of the dependence of the morphology, crystal structure, and luminescence properties of the resulting ceramics on the synthesis modes, electron energy, and electron flux power are presented.

## 1. Introduction

Materials based on metal oxides are widely used as dosimetric, scintillation, phosphors, and optical active media due to their high functional and operational properties [1,2,3,4]. It is possible to create many optical materials with complex compositions for various applications from metal oxides. It is possible to correct their properties by introducing modifiers, a combination of activators. Materials based on metal oxides of various structures are used: crystals, powders, and ceramics.

Most initial materials for material synthesis based on metal oxides are refractory; their melting points are usually in the range of 1000–4000 °C. Therefore, the synthesis of oxide materials is a difficult task. It is even more difficult to fulfill the conditions for the materials synthesis of complex compositions. Synthesis is realized through the exchange of elements of the initial substances, the melting points of which can vary greatly. Therefore, direct melting of initial materials to initiate element exchange reactions in the liquid state is possible only in very rare cases. For synthesis, it is necessary to use complex, multi-stage methods to use additional substances that contribute to the process. Various methods are used for the synthesis of YAG:Ce phosphors [5,6,7]. The most widespread and used in industry is the solid-phase reaction method of thermal synthesis and its modifications [8,9]. To implement the exchange of elements between the particles of the initial oxide powders Al_2_O_3_ (melting temperature t_m_ = 2044 °C), Y_2_O_3_ (t_m_ = 2410 °C), and Ce_2_O_3_ (t_m_ = 2177 °C) in a mixture of stoichiometric composition, synthesis is carried out in a liquid melt, for example, BaF_2_ (t_m_ = 1368 °C). With prolonged exposure at temperatures of 1400–1500 °C, particles stick together, and partial exchange of elements of different phases occurs. Then, at a higher temperature, BaF_2_ is removed by repeated annealing, and the formation of Y_3_Al_5_O_12_:Ce is completed. The synthesis procedure is complex, lengthy, and laborious. It is possible to obtain a good quality phosphor with good repeatability of luminescence properties, with strict observance of the synthesis regulations.

Other methods for the synthesis of luminescent materials based on YAG have been developed and are being improved. For example, the sol–gel method [10,11,12] and coprecipitation [13,14] produce ceramics and phosphors by using a combination of chemical reactions between substances containing the elements necessary for the formation of YAG. All these methods are complex; elements of the auxiliary materials used for the synthesis are preserved in the final products. They are interested in the synthesis methods of phosphors, ceramics in the flame of burners [15], and mixtures of combustible materials with metal oxide powders in approximately equal amounts [16]. Synthesis in the flame is realized in a short time. However, the synthesis process is difficult to control. Obviously, the resulting product may contain residues of combustible substances. It is assumed that it is possible to synthesize transparent ceramic materials by the SPS method (spark plasma sintering) [17].

For the first time, we proposed and implemented the synthesis method of YAG ceramics with activators from a mixture of Y_2_O_3_ and Al_2_O_3_ powders in the field of a powerful flux of high-energy electrons [18,19]. The method seems promising: the synthesis is realized in 1s, without the use of any substances facilitating the synthesis, with high productivity without the use of additional energy sources. It has been established that the set of processes ensuring high efficiency of radiation synthesis is completely different from those occurring when using other methods, in which the structure formation is stimulated by heat. It is assumed that the efficiency of synthesis in the field of powerful radiation streams is determined by the high density of ionization of matter and reactions in the electron–ion plasma.

There is an obvious need to establish the basic regularities of ceramics formation in the field of radiation of high density in order to optimize radiation exposure modes and to determine the requirements for the initial raw material, the processing methods of the resulting ceramics, expansion of the nomenclature of similar ceramics, etc. The possibility of express synthesis of materials contributes to the solution of these problems.

The present work is aimed at studying one of the most important tasks: studying the influence of radiation exposure modes on the initial mixture on the result and obtaining YAG ceramics with high functional properties.

It is necessary to study the dependence of the luminescence properties of this ceramic on various factors that may affect the result of synthesis. This article presents the results of such a study: the energy-transferring processes of the electron flux to the initial substance (mixture) to obtain YAG:Ce ceramics; the dependence of the synthesis result (morphology) on the synthesis modes, electron energy, and electron power flux; and the influence of synthesis modes on the crystal structure of the emerging ceramic’s luminescence properties.

## 2. Materials and Methods

### 2.1. Energy Losses of the Electron Beam in the Material

The distribution of absorbed energy in matter under the action of a spatially limited beam of high-energy electrons is inhomogeneous. The maximum absorbed energy is concentrated along the beam axis. The inhomogeneity of the distribution is even more pronounced when using beams with a Gaussian power distribution over the cross-section.

**Figure 1:**
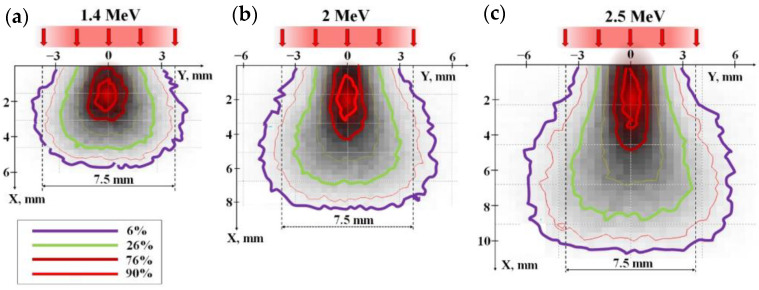
Energy loss distribution of electrons with E = 1.4 (**a**), 2.0 (**b**), 2.5 (**c**) MeV in a mixture with a bulk density of 1.2 g/cm^3^ for the synthesis of Y_3_Al_5_O_12_ ceramics. Colored lines of equal loss are given in units relative to the loss in the center.

Figure 1 defines the distribution profiles of electron losses calculated using the CASINO V2.5 program during their passage through a mixture with a bulk density of 1.2 g/cm^3^ from Y_2_O_3_ (57%) and Al_2_O_3_ (43%) powders used for the synthesis of Y_3_Al_5_O_12_ ceramics. The calculations were carried out by the Monte Carlo method for beams with a Gaussian flux income over the cross-section and velocity of incident electrons, with a density of 10,000, at energies of 1.4, 2.0, and 2.5 MeV, as used in experiments.

The electron beam entering the material is scattered by atoms and ions of the substance and transfers its energy to ionization and generation of secondary electrons. As a result of these processes, as the electrons pass through the substance, the spatial structure of the beam energy transfer changes. Part of the energy is transferred to matter outside the beam section. There is a concentration of energy losses along the beam axis. About 50% of the loss of the entire beam energy falls on the charge region along the beam axis with a cross-section of 0.3–0.4 of the beam area on the surface. This leads to a characteristic loss distribution along the beam axis for an electron beam.

**Figure 2:**
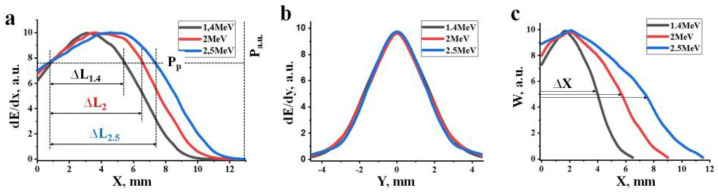
Energy loss distribution profiles dE/dx (**a**) and dE/dy (**b**) of electrons with energies of 1.4, 2.0, and 2.5 MeV in the mixture and absorbed energy density W (**c**).

Figure 2a shows the calculated profiles of the distribution of electron energy losses dE/dx in the mixture over depth for an equal number of incident electrons with energies of 1.4, 2.0, and 2.5 MeV. Here, the energy losses of the electrons in an electron beam during their passage through a substance are understood as the magnitude of losses at a depth X in the entire region perpendicular to the beam axis. The maximum of the absorbed energy is located at a certain depth from the surface, which depends on the energy of the electrons. The positions of the energy loss maxima dE/dx of the beams fall at 2.8, 3.7, and 4.6 mm for the indicated electron energies. The value of energy losses in the maxima is 30–40% higher than at the target surface.

Figure 2b shows the calculated distribution profiles of electron energy losses dE/dy in the direction perpendicular to the beam axis. It can be seen from the results of calculating the energy loss distribution of electrons of different energies presented in Figure 1 that the profiles dE/dy change with depth. These changes are different for electron beams of different energies. The figure shows the profiles dE/dy for the depths corresponding to the maximum absorbed energy density Wr of the electron beam. The absorbed energy density W is understood as the amount of energy loss per unit volume of a substance. The dE/dy profiles match. This means that under the condition adopted for the calculation, an equal number of incident electrons, the values of Wr are the same.

The energy loss densities along the axis of the passing beam are always higher than outside the axis and have the form of a curve with a maximum. Figure 2 c shows the calculated profiles of the dependence of W on the depth of electron passage. Dependences have the form of curves with maxima at 1.8, 2.1, and 2.3 mm for electrons with energies of 1.4, 2.0, and 2.5 MeV. The region length Δx with an equal density of absorbed energy along the axis increases on average by a factor of 2 with an increase in the electron energy from 1.4 to 2.5 MeV. Note that the energy loss maxima dE/dx are located at depths of 2.8, 3.7, and 4.6 mm at energies E equal to 1.4, 2.0, 2.5 MeV, and the region length with the same absorbed energy along the axis with increasing electron energy from 1.4 to 2.5 MeV increases by only 25% on average. This is due to the fact that by the end of the run, an expansion of the energy loss region is observed. Thus, the maxima of energy loss in depth (Figure 2a) (dE/dx) and energy loss densities W (Figure 2c) do not coincide.

Obviously, the areas of maximum energy loss densities should determine subsequent processes. It is in these areas that the ionization density is maximum, and the temperature to which the material is heated is maximum. In these regions, during the time of beam exposure, when the energy loss exceeds the threshold value, the crystal structure of yttrium and aluminum oxides is transformed into yttrium aluminum garnet. First of all, synthesis should be carried out at depths corresponding to the maximum energy loss densities along the beam axis, but not energy losses.

With a change in the electron beam power P, the distribution profile does not change, but the absolute values of the energy losses dE/dx and dE/dy change proportionally. Synthesis is realized in the material when the energy losses dE/dx and dE/dy in a specific region of the material with XY coordinates exceed a certain threshold value P_p_ of the beam power. The threshold P_p_ at which the synthesis can be realized depends only on the composition of the initial mixture, that is, on the composition of the synthesized material. Synthesis may not occur at the target surface and at great depths, but it may occur in the depth range ΔL, at which the energy loss exceeds the value required for synthesis. The range of depths ΔL at which synthesis can be realized increases with increasing E. Synthesis is realized in the depth range in which the energy loss density W exceeds the value required for synthesis. With an increase in the electron beam power P, the length increases, and the diameter of the region of maximum energy loss W along the beam axis increases. Therefore, synthesis can be realized in a larger volume, in which the energy loss densities exceed the synthesis realization threshold P_p_.

Thus, the volumetric energy loss density changes significantly in the longitudinal and transverse to the direction of propagation of the electron flux in the substance. With an increase in the power density of the input electron flux, the energy losses increase proportionally. In the region of maximum energy losses, the process of material synthesis is most likely to be realized. With an increase in the power of the electron beam, the volume of the material in which synthesis can be realized increases, the upper limit of this volume can reach the surface of the mixture, and the lower limit can reach depths equal to the length of the extrapolated electron path Xe. The extrapolated depth of the free path of electrons in the mixture for the synthesis of YAG:Ce ceramics (see Figure 2a) is 9, 10, and 11 mm for electrons of 1.4, 2.0, and 2.5 MeV, respectively. 

### 2.2. Synthesis of YAG Ceramics

A cycle of studies of the efficiency dependence of radiation synthesis of YAG:Ce ceramics on the electron energy and beam power was completed. The concentration of Ce introduced for activation was 0.5%. Such an amount of the activator does not affect the main regularities of energy losses but allows using luminescence methods to make sure that Ce is included in the crystalline structure of ceramics. The prepared mixture with the composition of Y_2_O_3_ (56%), Al_2_O_3_ (43%), and Ce_2_O_3_ (0.5% by weight of the mixture) had a bulk density of 1.2 g/cm^3^. Synthesis of YAG:Ce ceramics was carried out in copper crucibles with a depth of 14 mm, exceeding the total electron path at E = 2.5 MeV and dimensions of 50 × 100 mm.

Synthesis was carried out by direct action on the mixture in the crucible of an electron beam with energies of 1.4, 2.0, and 2.5 MeV extracted into the atmosphere from the ELV-6 accelerator of the INP named after. Budker SB RAS. The electron beam with a Gaussian flux distribution had a diameter of 1 cm on the mixture surface. Two modes of action of the electron beam on the mixture were used: “without scanning”, when the crucible was pulled relative to the beam; and “with scanning”, when the crucible with dimensions of 50 × 100 mm was pulled relative to the beam scanning in the transverse direction at a frequency of 50 Hz. The crucible was stretched along its entire length under the electron beam for 10 s. To obtain equality of the absorbed energy, the beam power in the “with scanning” mode was 5 times greater than in the “without scanning” mode.

Since the distribution of the absorbed energy of an electron beam in a substance is heterogeneous, it is necessary to designate the criteria for choosing the irradiation conditions under which the comparison of the results of synthesis by beams with different energies will be correct. The densities of maximum beam energy losses in the irradiated region of the substance should be close. Our previous studies have shown that when using an electron beam with E = 1.4 MeV, the synthesis of YAG:Ce ceramics in the “without scanning” mode is successfully implemented when transferring a charge with a bulk density of 1.2 g/cm^3^ to a substance with an energy of 4 kJ/s cm^3^ in the central region, to which 50% of the absorbed energy is transferred. Such an absorbed energy density is provided under the used irradiation conditions by a beam with a power of 5 kW/cm^2^. When irradiated with electrons with higher energies, 50% of the absorbed energy at the center of the beam passage occurs in a larger volume. Based on the study of the dependence of the absorbed energy distribution on the electron energy (Figure 1 and Figure 2), we have shown that the electron beam power should be 1.4 times higher for electrons with E = 2.0 MeV and 1.8 times higher for electrons with E = 2.5 MeV. The correction of modes during synthesis was carried out experimentally.

**Figure 3:**
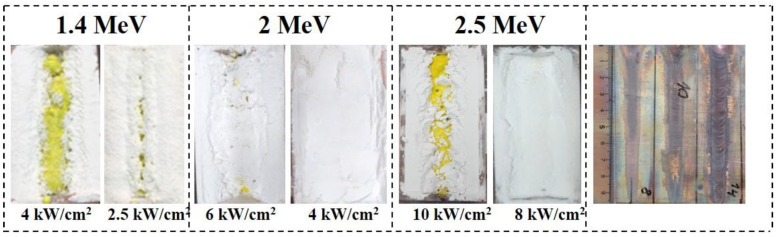
Photographs of ceramic samples synthesized under the exposure to electron fluxes with E = 1.4 MeV (P = 4 and 2.5 kW/cm^2^), E = 2.0 MeV (P = 6 and 4 kW/cm^2^), and E = 2.5 MeV (P = 10 and 8 kW/cm^2^), and traces of the impact of electron flows with E = 1.4 MeV (P = 8, 10, 14 kW/cm^2^) on the copper plate.

Photographs of ceramic samples in crucibles synthesized under the influence of electron fluxes with E = 1.4 MeV, E = 2.0 MeV, and E = 2.5 MeV at different power densities P are shown in Figure 3. The synthesis was carried out in the “without scanning” mode, which makes it possible to visually compare the results of the analysis of energy losses and synthesis.

It can be seen from the above images that during the exposure time of 10 s, electron beams form ceramic samples in the mixture in the form of rods of yellow color characteristic of YAG:Ce. At large P, the rod samples are on the irradiated surface or close to the surface. With a decrease in P, the formed samples can be hidden under a layer of mixture. The depth of the formed sample in the mixture is greater the higher the value of E. This regularity corresponds to the conclusion made above about the dependence of the position of the region of maximum energy loss of the electron beam on E and P. 

The same figure shows photographs of traces of the impact of an electron beam with E = 1.4 MeV in the “without scanning” mode on a thick copper plate. In the experiment, the upper surface of the plate was placed at the same distance from the accelerator outlet, where the outer surface of the charge was located during synthesis. The images clearly show that the width of the trace of the impact of the flux in the middle of the image reaches 7–10 mm, and the flow power in the center is much higher. At a flux density of P = 8 kW/cm^2^, only a trace of oxidation is visible in the image; at P = 12kW/cm^2^, melting of the outer surface is observed. We emphasize that the melting temperature of copper (1085 °C) is used for the synthesis of oxides: Al_2_O_3_ (2044 °C) and Y_2_O_3_ (2410 °C). The synthesis of YAG:Ce ceramics is realized under the same conditions of exposure to an electron beam at P < 4 kW/cm^2^, which is explained by the difference in the processes of dissipation of the absorbed energy of hard radiation in metals and dielectrics.

We also note the following effect. The photographs shown in Figure 3 show that the synthesis of YAG:Ce ceramics is realized almost uniformly along the entire length of the crucible, which is moved relative to the electron beam. The trace of the impact of the electron beam on the copper plate has a variable width. As the plate moves (or with time after the beginning of the beam impact), the trace expands. The expansion of the track is due to the fact that, over time, the temperature of the entire volume of the copper plate increases due to its high thermal conductivity (401 W/m* C). The thermal conductivity of the mixture for synthesis is 0.15–0.16 W/m* C, three orders of magnitude lower than in copper [19,20]. The time of passage of the irradiated section of the charge is 1 s. During this time, heat from the irradiated area does not have time to be transferred to the environment [17].

**Figure 4:**
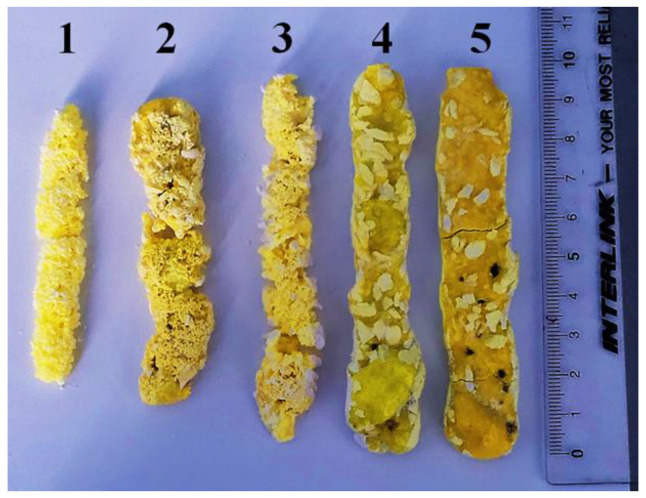
Photographs of YAG:Ce ceramic samples synthesized under the influence of electron fluxes of different E and P: 1—E = 1.4 MeV, P = 2.5 kW/cm^2^; 2—E = 2.0 MeV, P = 4 kW/cm^2^; 3—E = 2.5 MeV, P = 8 kW/cm^2^; 4—E = 2.0 MeV, P = 6 kW/cm^2^; 5—E = 2.5 MeV, P = 10 kW/cm^2^.

Figure 4 shows photographs of the samples taken out of the crucibles. The first three were completely covered by the mixture; the last two were open. All samples have the form of rods of different diameters. Sample 1 was synthesized at E = 1.4 MeV, P = 2.5 kW/cm^2^; sample 2 at E = 2.0 MeV, P = 4 kW/cm^2^; and sample 3 at E = 2.5 MeV, P = 8 kW/cm^2^. Sample 4 was only slightly covered by the mixture from above (E = 2.0 MeV, P = 6 kW/cm^2^), whereas sample 5 was almost completely open (E = 2.5 MeV, P = 10 kW/cm^2^).

The samples formed inside the mixture, at a relatively low power density P, have a shorter length and a porous surface. Ceramic samples that reached the surface of the mixture during the formation of ceramics have a solid surface but are porous inside. Note that the light spots in the photographs of the samples are the mixture traces, which are difficult to remove without damaging the sample. 

As P decreases, the diameter of the forming sample decreases, and the solid rod turns into a dotted one. The smallest ceramic samples in the form of rare dotted particles with sizes of about 3 mm in diameter and up to 10 mm in length were obtained by exposure to electron beams with E = 1.4 MeV and P = 1.5 kW/cm^2^. The samples are friable and crumble under slight pressure. However, they have a characteristic yellow color for YAG:Ce ceramics. 

**Figure 5:**
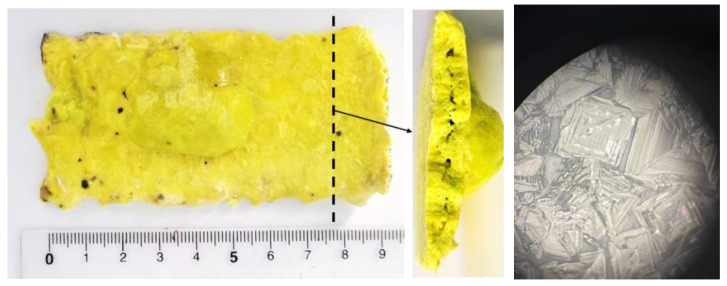
Photographs of YAG:Ce ceramic sample synthesized at E = 2.5 MeV, P = 37 kW/cm^2^. On the right is a photograph of the outer surface of the ceramic sample taken by optical microscope.

Figure 5 shows a photograph of a YAG:Ce sample synthesized by the “scanned” method at E = 2.5 MeV, P = 37 kW/cm^2^. The sample has the form of a plate with dimensions of 90 × 45 mm. The plate surface is uneven. The sample thickness is 6 mm on average. The weight of the plate is 83 g. Inside the plate is porous, but the porosity is much lower than that of the samples obtained by the “without scanning” method at the same electron energy and absorbed energy. The pores are large and located parallel to the outer surfaces of the plate. The thickness of dense layers is 2–2.5 mm. The porosity of samples obtained at lower E and P is much higher.

A visual representation of the synthesized ceramic’s surface structure is given in a photograph taken by an optical microscope, the “XJP 146 Trinocular Microscope” (Ningbo Wason Optical Instrument Co., Ltd., Zhejiang, China ). The outer surface image taken by the optical microscope is shown in Figure 5 on the right. The surface looks like a set of regular shape crystallites bound together by a binding phase. The surface layer is hard but brittle. When an indenter is pressed, the surface breaks. The crystallites are up to 0.1 mm in size.

**Figure 6:**
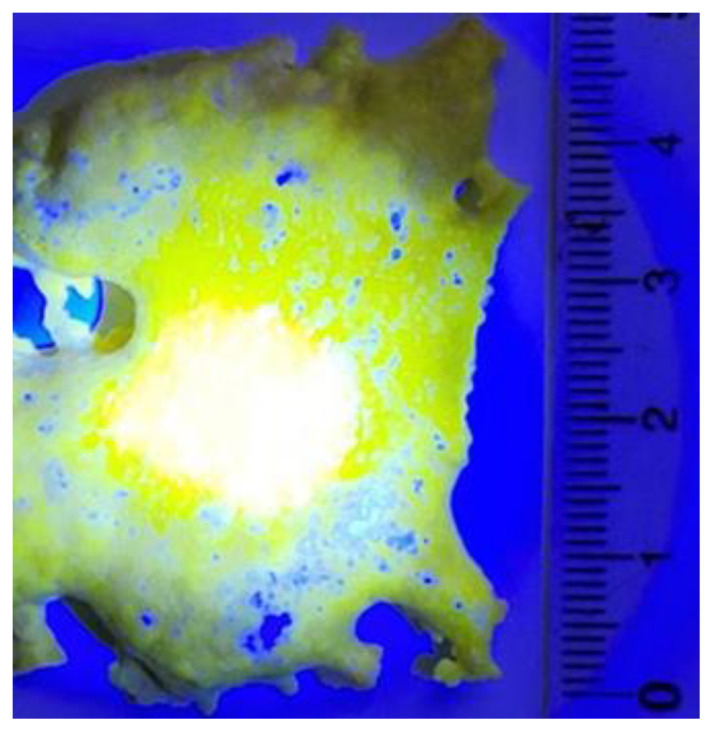
Photo of YAG:Ce ceramic sample synthesized at E = 1.4 MeV, P = 25 kW/cm^2^, illuminated by chip radiation λ = 450 nm.

Figure 6 shows a photograph of a part of a YAG:Ce ceramic sample synthesized by the “scanned” method at E = 1.4 MeV, P = 25 kW/cm^2^. The sample has the form of a plate with uneven edges, with a total area of 80 × 45 mm. The plate has a variable area thickness from 0 to 5 mm. The cross-section of the ceramic is more porous than that obtained at E = 2.5 MeV. The thickness of the dense layers near the surfaces is 1–1.5 mm.

The photo was taken when the sample was illuminated by a concentrated lens with chip radiation with λ = 450 nm. This radiation excites the luminescence of YAG:Ce ceramics. The white color of the central part of the sample is due to luminescence and reflected radiation from the chip. The near-yellow color is due to the reflection of the white light of the central region by the ceramics. The blue color is due to the reflection of the chip radiation from the white paper on which the sample was placed.

## 3. Results

### 3.1. Structure of Synthesized Ceramics

**Figure 7:**
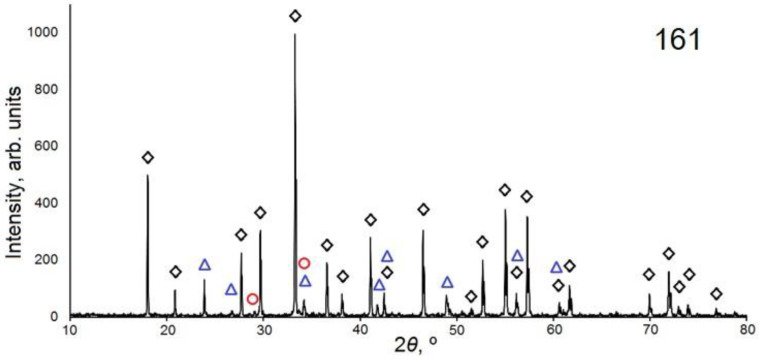

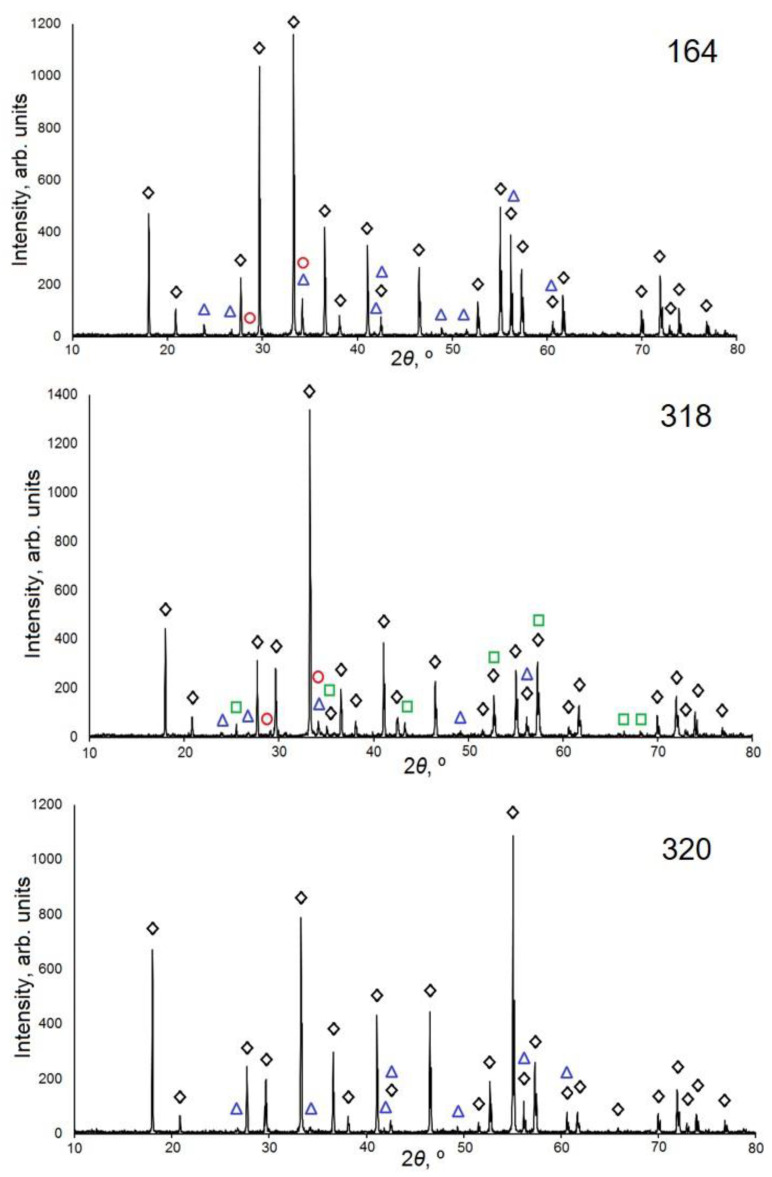

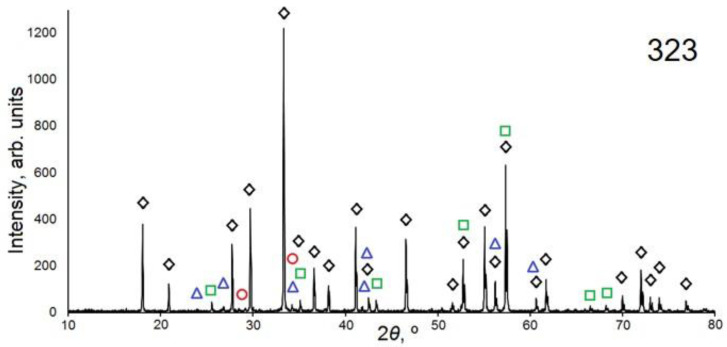
X-ray diffraction patterns of Y_3_Al_5_O_12_ ceramic samples. Designations: black rhombus—Y_3_Al_5_O_12_ reflections, blue triangle—YAlO_3_, red circle—Y_2_O_3_, green square—Al_2_O_3_, black star—Y_4_A_l2_O_9_. The serial number in the figures is the sample number in the accounting system used by the authors.

The structure of the synthesized ceramics was studied by X-ray diffraction using a D8 ADVANCE Bruker diffractometer equipped with a scintillation detector in a step-by-step shooting mode in the range of diffraction angles 2θ from 10 to 80 degrees using a CuKα radiation source. The experiments were carried out at room temperature in the Bragg–Brentano geometry with a flat sample. The experimental conditions were as follows: 40 kV, 40 mA, exposure time—1 s, and step size—0.02° 2θ. The received data were processed using the DIFFRACplus software package. Samples were identified using the PDF-2 Powder Diffraction Database (ICDD, 2007) and indexed using EVA software (Bruker, 2007). In the TOPAS 4 program (Bruker, 2008), using the Rietveld method, the parameters of the YAG unit cell and the relative content of the main and accompanying phases were refined. The phase detection limit and the error for semi-quantitative analysis are 1–3% and 1–5%, respectively. The diffraction patterns of the samples are shown in Figure 7.

For qualitative phase analysis and identification of diffraction patterns, the following data from the PDF-2 file (ICDD, 2007) were used: PDF 00-033-0040 “Aluminum Yttrium Oxide (Al_5_Y_3_O_12_)”, PDF 01-070-1677 “Yttrium Aluminum Oxide (YAlO_3_)”, PDF 00-046-1212 “Aluminum Oxide (Al_2_O_3_)”, PDF 00-041-1105 “Yttrium Oxide (Y_2_O_3_)”, and PDF 01-083-0933 “Aluminum Yttrium Oxide (Al_2_Y_4_O_9_)”.

The results of studying the phase composition of the samples are shown in Table 1.

Samples 161 and 164 are almost identical in their phase composition: they contain YAG and accompanying phases (YAlO_3_ and Y_2_O_3_) in approximately the same ratio. The powder diffraction patterns of samples 318 and 323, in addition to those listed above, also have Al_2_O_3_ as an accompanying phase and, at the same time, are very close to each other in composition. The purest sample is YAG 320, which contains about 3% YAlO_3_ impurities (~3%). In sample 161, the amount of the YAlO_3_ phase is 7%. The obtained unit cell parameters for Y_3_Al_5_O_12_ in all samples are very close (Δa = 0.006 Å).

Thus, the synthesized ceramics have the Y_3_Al_5_O_12_ phase as the main one. The proportion of this phase is the same in all samples obtained using electron energies of 1.4, 2.0, and 2.5 MeV and flux powers (in the “without scanning” mode) from 2.5 to 14 kW/cm^2^.

### 3.2. Luminescence of Synthesized Ceramics

**Figure 8:**
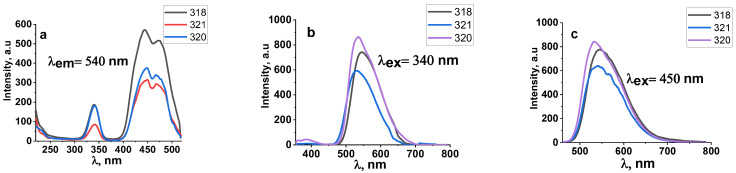
Excitation (**a**) and luminescence spectra (**b**,**c**) of ceramic samples synthesized under the exposure to an electron beam with E = 2.5 MeV and P = 8 and 10 kW/cm^2^ in the “without scanning” mode.

**Figure 9:**
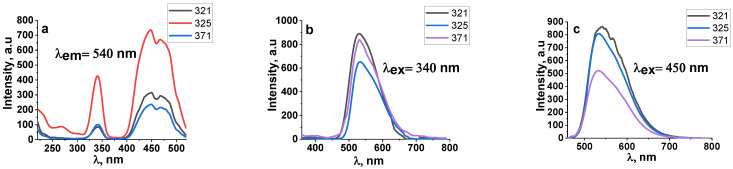
Excitation (**a**) and luminescence (**b**,**c**) spectra of ceramic samples synthesized when exposed to an electron beam with E = 1.4, 2.0, 2.5 MeV and P = 25, 33, 37 kW/cm^2^ in “with scanning” mode.

A series of studies of the luminescence properties of YAG:Ce ceramics synthesized under different irradiation modes has been carried out. The synthesis was carried out at electron energies of 1.4, 2.0, and 2.5 MeV and electron beam powers in the range from 2.5 to 14 kW/cm^2^ (in the “without scanning” mode) and from 12 to 40 kW/cm^2^ (in the “with scanning” mode). We measured the excitation and luminescence spectra under stationary conditions at room temperature using an Agilent Cary Eclipse spectrofluorometer for all YAG:Ce ceramic samples synthesized under different irradiation conditions. Examples of spectra measured in samples synthesized with different E and P are shown in Figure 8 and Figure 9. Sample synthesis modes are shown in Table 2. 

Figure 8 shows the excitation and luminescence spectra of ceramic samples synthesized under exposure to an electron beam with E = 2.5 MeV and P = 8 and 10 kW/cm^2^ in the “without scanning” mode. The spectra are similar. Additionally, the excitation and luminescence spectra of ceramic samples synthesized under exposure to an electron beam with E = 2.5 MeV and P = 37 kW/cm^2^ in the “with scanning” mode are shown. At E = 2.5 MeV and P = 8 kW/cm^2^ in the “without scanning” mode and P = 37 kW/cm^2^ in the “with scanning” mode, the maximum values of the absorbed energy density Wr are equal. The difference is that in the “without scanning” mode, each irradiated area of the substance is exposed to the Gaussian electron beam passing through this area. In the “with scanning” mode, each area of the substance is exposed to a series of pulses of a scanning beam with a frequency of 50 Hz and a duration of 2 ms with a Gaussian envelope. The excitation and luminescence spectra of all measured samples are similar. Consequently, under the specified synthesis conditions, a change in the power and method of introducing the beam energy into the substance does not affect the entry of the activator into the crystal structure of ceramic crystallites.

Figure 9 shows the excitation and luminescence spectra of ceramic samples synthesized under exposure to an electron beam with E = 1.4, 2.0, and 2.5 MeV and P = 25, 33, and 37 kW/cm^2^ in the “with scanning” mode. At the indicated powers P of the electron beam, the maximum values of the absorbed energy density W_r_ are equal.

As can be seen from the presented measurement results, the luminescence excitation spectra of ceramic samples synthesized when exposed to an electron beam of different energies are similar. In particular, the luminescence spectra upon excitation in the bands with radiation at 340 and 450 nm are similar. Note that the spectra in Figure 8 and Figure 9 are completely similar to those published in many works devoted to the study of the spectral characteristics of YAG:Ce phosphors and ceramics and are explained by the existence of absorption and emission levels in cerium ions [21,22,23]. It is assumed that two Ce^3+^ excitation bands at 460 and 340 nm are due to ^4^F_5/2_ → ^5^D_0_, ^5^D_1_ transitions, and the broad luminescence band at 520 and 580 nm is due to ^5^D_0_ → ^4^F_5/2_, ^4^F_7/2_ transitions.

Thus, the radiation synthesis modes, the power of the high-energy electron beam, and their energy do not affect the spectral luminescence characteristics of the resulting ceramics.

## 4. Discussion

Electron beams are widely used for spraying, sputtering materials [24,25], melting refractory materials [26,27], property modification [28,29], creating coatings [30], and making nanopowders [31].

The promising use of electron beams is due to the high efficiency of conversion of the input energy into the energy of the electron beam, the efficiency of energy transfer to the workpiece and surface, the simplicity of beam control, and the purity of processing procedures. Most frequently, electron fluxes with energies in the range of 10^2^–10^5^ eV are used for this purpose. For sterilization and introscopy, electron fluxes with energies up to 10 MeV are used. Of particular interest is the use of electrons with energies between 1.0 and 3.0 MeV. Electron accelerators of such energies are simple, although large-sized devices capable of generating electrons with flux power up to 100 kW, which allows the instantaneous transfer of high energy density to matter. Such accelerators have found application in the creation of multi-layer metal articles of great thickness.

We have shown the possibility of obtaining, using such electron beams, dielectric materials with new structural phases from initial substances with the same elemental composition. In the field of a high-energy electron flux, YAG ceramics were obtained from Y and Al oxides with the desired stoichiometric composition. It turned out that the synthesis of high-temperature (refractory) YAG ceramics from Al_2_O_3_ (t_m_ = 2044 °C) and Y_2_O_3_ (t_m_ = 2410 °C). It has been established that this effect is explained by the dominant role of ionization processes in dielectric materials and their relaxation after creation. Therefore, it is extremely important to understand the elementary processes of electron flux energy transfer to matter.

A quantitative analysis of the energy transfer of an electron beam with a spatially limited cross-section with energies of 1.4, 2.0, and 2.5 MeV has been performed. It is shown that the beam energy is transferred to the substance inhomogeneously. In a mixture of Y_2_O_3_ (57%) and Al_2_O_3_ (43%) powders with a bulk density of 1.2 g/cm^3^ used for the synthesis of Y_3_Al_5_O_12_ ceramics, the energy maximum dE/dx is transferred to the substance along the beam propagation axis at a depth of 2.8, 3.7, and 4.6 mm at extrapolated electron path lengths of 9, 10, and 11 mm at energies E equal to 1.4, 2.0, and 2.5 MeV. At these depths, the diameter of the energy loss cross-sectional area exceeds 8, 10, and 12 mm, respectively.

The maxima of the absorbed energy density W at E = 1.4, 2.0, and 2.5 MeV are located along the beam axis and are at depths of 1.8, 2.1, and 2.3 mm. In these areas, the ionization density, the temperature to which the material is heated, is maximum. Obviously, the regions of maximum absorbed energy densities W determine the subsequent processes of structural transformations. In these areas, during the exposure to the beam, energy loss densities sufficient to transform the crystal structure of yttrium and aluminum oxides into yttrium aluminum garnet can be achieved. 

The absorbed energy density W is proportional to the electron beam power density P. Structural transformations are realized when P exceeds the threshold P_p_. As P increases above P_p_, the range ΔL increases (Figure 2a), which can reach ΔL_e_, the extrapolated electron range. The distribution of the absorbed energy density W and the position of the maximum absorbed energy density W_r_ depend on the electron energy, as shown in Figure 2c.

For experimental studies of the synthesis dependence on E and P, a series of samples with the same composition of Y_2_O_3_ (57%) and Al_2_O_3_ (43%) + Ce_2_O_3_ (0.5% of the total mass) was obtained. The P ranges (without scanning) were chosen as 1.5–6, 4–8, and 8–10 kW/cm^2^ for E = 1.4, 2.0, and 2.5 MeV, respectively, which fit the W ratios expected from the modeling results. Synthesis under all irradiation modes was realized at a rate above 1 cm/s, completely—in the entire crucible—for 10 s. In all modes, samples of YAG:Ce ceramics of a characteristic yellow color were obtained. At low P, the samples in the form of rods obtained in the mode (without scanning) were covered from above with a layer of charge. As P increased, specimens with an open surface and greater thickness were obtained. As P decreases, the thickness of the forming sample decreases, and the solid rod turns into a dotted one. The smallest samples of ceramics in the form of rare dotted particles with sizes of about 3 mm in diameter and up to 10 mm in length were obtained by exposure to electron beams with E = 1.4 MeV and P = 1.5 kW/cm^2^ (without scanning mode). The samples are friable and crumble under slight pressure. These dimensions, apparently, can be considered the smallest, below which radiative synthesis does not occur. The largest dimensions of the YAG:Ce ceramic sample were obtained at E = 2.5 MeV and P = 37 kW/cm^2^ (“with scanning” mode). The sample has the form of a plate with dimensions of 90 × 45 mm; the plate weight is 83 g.

The results of the XRD study showed that the dominant phase in the obtained samples was Y_3_Al_5_O_12_, and the accompanying phase was the YAlO_3_ phase in an amount from 3 to 7%. The proportion of the main Y_3_Al_5_O_12_ phase exceeds 90% in all samples obtained using electron energies of 1.4, 2.0, and 2.5 MeV and flux powers (in the “without scanning” mode) from 2.5 to 14 kW/cm^2^.

The evidence for the formation of YAG:Ce ceramics is the results of studying the luminescence spectra of the synthesized samples. All synthesized samples of YAG:Ce ceramics have characteristic luminescence bands at 540 nm and excitation bands at 340 and 450 nm in their spectra. Consequently, the activator ions, in a short synthesis time of less than 1 s, have time to integrate into the nodes of the emerging lattice.

## 5. Conclusions

This paper presents the results of optimal modes search of radiation synthesis of dielectric materials by the example of YAG:Ce ceramics synthesis. Based on the conducted research, the flux rates in the ranges of 3–5, 5–8, and 7–12 kW/cm^2^ (“without scanning” mode) using electron energies of 1.4, 2.0, and 2.5 MeV are optimal for the radiation synthesis of YAG:Ce ceramics. The use of E > 3–4 MeV electrons for synthesis may allow for obtaining samples of greater thickness. However, synthesis with electrons of these energies requires a significant increase in power density to compensate for the decrease in the average ionization density due to the increase in the volume of the energy loss region. When using electrons with E < 0.5 MeV, the region of high ionization density becomes so narrow that part of the absorbed energy will go beyond the optimal synthesis region. It should be emphasized that the conclusions and patterns presented are characteristic of dielectric materials only. In metals, the absorbed energy of radiation flux is immediately transferred to the lattice and leads to material heating. Due to the high thermal conductivity of metals, which is two orders of magnitude higher than in dielectrics and three orders of magnitude higher than in dielectric powders, the absorbed energy quickly leaves the region with the maximum absorbed energy density. In metals, only processes stimulated by radiation heating of the material are initiated.

## Figures and Tables

**Table 1:** Results of studying the phase composition of the samples.

	Main Phase	Related Phase	Rwp(%)
161	Y_3_Al_5_O_12_ (~91%) *Ia*–3*d*; *a* = 12.005(2) Å; *V* = 1730.3(3) Å^3^	YAlO_3_ (~7%) Y_2_O_3_ (~2%)	5.0
164	Y_3_Al_5_O_12_ (~92%) *Ia*–3*d*; *a* = 12.009(4) Å; *V* = 1732.1(3) Å^3^	YAlO_3_ (~6%) Y_2_O_3_ (~2%)	5.6
318	Y_3_Al_5_O_12_ (~91%) *Ia*–3*d*; *a* = 11.999(2) Å; *V* = 1727.4(3) Å^3^	YAlO_3_ (~4%) Al_2_O_3_ (~3%) Y_2_O_3_ (~2%)	4.3
320	Y_3_Al_5_O_12_ (~97%) *Ia*–3*d*; *a* = 12.002(2) Å; *V* = 1728.9(3) Å^3^	YAlO_3_ (~3%)	5.0
323	Y_3_Al_5_O_12_ (~90%) *Ia*–3*d*; *a* = 12.005(2) Å; *V* = 1730.1(3) Å^3^	YAlO_3_ (~5%) Al_2_O_3_ (~3%) Y_2_O_3_ (~2%)	5.3

**Table 2:** Synthesis modes. The serial number in the table is the sample number in the accounting system used by the authors.

No.	E, MeV	P, kW	
318	2.5 MeV	8 kW	Without Scan
320	2.5 MeV	10 kW	Without Scan
321	2.5 MeV	37 kW	With Scan
325	2.0 MeV	30 kW	With Scan
371	1.4 MeV	25 kW	With Scan

## Data Availability

The data presented in this study are available on request from the corresponding author.
